# A living scoping review and online repository of artificial intelligence models in pediatric urology: Results from the AI-PEDURO collaborative

**DOI:** 10.1016/j.jpurol.2025.01.035

**Published:** 2025-02-05

**Authors:** Adree Khondker, Jethro CC. Kwong, Ihtisham Ahmad, Zwetlana Rajesh, Rahim Dhalla, Wyatt MacNevin, Mandy Rickard, Lauren Erdman, Andrew T. Gabrielson, David-Dan Nguyen, Jin Kyu Kim, Tariq Abbas, Nicolas Fernandez, Katherine Fischer, Lisette A. t Hoen, Daniel T. Keefe, Caleb P. Nelson, Bernarda Viteri, Hsin-Hsiao (Scott) Wang, John Weaver, Priyank Yadav, Armando J. Lorenzo

**Affiliations:** a Division of Urology, The Hospital for Sick Children, Toronto, ON, Canada; b Division of Urology, Department of Surgery, University of Toronto, Toronto, ON, Canada; c Temerty School of Medicine, University of Toronto, Toronto, ON, Canada; d Schulich School of Medicine, University of Western Ontario, London, ON, Canada; e Department of Urology, IWK Hospital, Halifax, NS, Canada; f Temerty Centre for AI Research and Education in Medicine, University of Toronto, Toronto, ON, Canada; g James M. Anderson Center for Health Systems Excellence, Cincinnati Children’s Hospital Medical Center and University of Cincinnati School of Medicine, Cincinnati, OH, USA; h James Buchanan Brady Urological Institute, Johns Hopkins University School of Medicine, Baltimore, MD, USA; i Institute of Health Policy, Management and Evaluation, Dalla Lana School of Public Health, University of Toronto, Toronto, ON, Canada; j Division of Urology, Riley Children’s Hospital, Indianapolis, IN, USA; k Division of Urology, Sidra Medicine, Doha, Qatar; l Division of Pediatric Urology, Seattle Children’s Hospital, Seattle, WA, USA; m Division of Urology, Children’s Hospital of Philadelphia, Philadelphia, PA, USA; n Department of Urology, Sophia Children’s Hospital, Erasmus University Medical Center, Rotterdam, the Netherlands; o Department of Urology, Boston Children’s Hospital, Boston, MA, USA; p Division of Nephrology, Children’s Hospital of Philadelphia, Philadelphia, PA, USA; q Department of Urology, Cleveland Clinic Children’s, Cleveland, OH, USA; r Division of Urology, Sanjay Gandhi Institute of Medical Sciences, Lucknow, India

**Keywords:** Artificial intelligence, Machine learning, Pediatric urology, Prediction, Model quality assessment

## Abstract

**Introduction:**

Artificial intelligence (AI) is increasingly being applied across pediatric urology. We provide a living scoping review and online repository developed by the AI in PEDiatric UROlogy (AI-PEDURO) collaborative that summarizes the current and emerging evidence on the AI models developed in pediatric urology.

**Material and methods:**

The protocol was published a priori, and Preferred Reporting Items for Systematic Review and Meta-analysis Scoping Review (PRISMA-ScR) guidelines were followed. We conducted a comprehensive search of four electronic databases and reviewed relevant data sources from inception until June 2024 to identify studies that have implemented AI for prediction, classification, or risk stratification for pediatric urology conditions. Model quality was assessed by the APPRAISE-AI tool.

**Results:**

Overall, 59 studies were included in this review from 1557 unique records. Of the 59 published studies, 44 studies (75 %) were published after 2019, with hydronephrosis and vesicoureteral reflux/urinary tract infection as the most common topics (17 studies, 28 % each). Studies originated from USA (22 studies, 37 %), Canada (10 studies, 17 %), China (8 studies, 14 %), and Turkey (7 studies, 12 %). Neural network (35 studies, 59 %), support-vector-machine (21 studies, 36 %), and tree-based models (19 studies, 32 %) were the most used machine learning algorithms, with 14 studies (24 %) providing useable repositories or applications. APPRAISE-AI assessed 12 studies (20 %) of studies as low quality, 39 studies (66 %) as moderate quality, and 8 studies (14 %) as high quality, with specific improvements noted in model robustness and reporting standards over time (p = 0.03). Findings were synthesized into an online repository (www.aipeduro.com).

**Discussion:**

There is an increasing pace of AI model development in pediatric urology. Model topics are broad, algorithm choice is diverse, and the overall quality of models are improving over time. While there is still a lack of clinical translation of the AI models in pediatric urology, the usage of online repositories and reporting frameworks can facilitate sharing, improvement, and clinical implementation of future models.

**Conclusions:**

This living scoping review and online repository will highlight the current landscape of AI models in pediatric urology and facilitate their clinical translation and inform future research initiatives. From this work, we provide a summary of recommendations based on the current literature for future studies.

## Introduction

Artificial intelligence (AI) is among the most exciting topics in medicine and has a rich history in pediatric urology. Machine learning (ML) is the application of AI that focuses on algorithms that are capable of learning from data, while AI is the broader umbrella term that encompasses systems that and technologies that are designed to simulate human intelligence. Bagli et al. (1998) were the first to apply ML in pediatric urology, showing that ML models could predict post-operative outcomes in pediatric hydronephrosis [1]. Since then, over 40 publications have applied ML methods to predict personalized risk of urinary tract infection (UTI) [2], standardize hypospadias assessment [3,4], and identify patients who can safely avoid investigations in hydronephrosis [5,6], among many others [7,8]. These ML models improve diagnostic accuracy, uncover new clinical associations, help with prognosis, and ultimately enhance patient care. However, to be useful in practice, ML models need rigorous validation, clear governance, and careful deployment [9,10].

Three systematic evidence syntheses to date have highlighted a growing number of AI models in pediatric urology, addressed the knowledge gap between the urology and data science communities, and revealed that most models are of moderate quality according to current reporting standards [7,8,11]. Although these reviews show great promise, the models in pediatric urology are subject to unique limitations. Models are often developed on single-institutional studies, low patient numbers, and outcomes are based on evidence-based guidelines that rely on low quality evidence. For example, while the most common problem for AI in pediatric urology is pediatric hydronephrosis, management in this topic is based on expert opinion, non-randomized studies and subject to institutional variation [12,13]. To address these challenges, we need a shared database of AI models to improve transparency and collaboration between clinicians, computer scientists, and patients. A shared database would facilitate collaborative research, promote best practices, and enhance model validation. We aim to build this database with the AI-PEDURO (Artificial Intelligence in PEDiatric UROlogy) collaborative.

In this review, we present aims to synthesize the current evidence on AI models in pediatric urology and create an accessible online repository that fosters collaboration and knowledge sharing.

## Methods

### Review summary

The protocol for this scoping review was previously published [14]. The reporting of the scoping review followed the Preferred Reporting Items for Systematic Reviews and Meta-Analyses for Scoping Reviews (PRISMA-ScR) [15]. The objectives of this work were to:

- Identify current topics of interest where AI has been utilized and, conversely, which topics lack AI-based models.
- Analyze trends in model development, AI dissemination, and ML algorithm adoption in clinical practice.
- Appraise the quality and risk of bias using standard ized and validated methods with current AI models.

### Search strategy

A detailed search strategy was developed for four electronic databases. MEDLINE (via Ovid), EMBASE (via Ovid), Scopus, and CINAHL (EBSCO) were searched in June 2024. A detailed search strategy and corresponding search terms are provided in Supplementary Table 1. The search was not restricted by language and from database conception to June 2024. Studies were also taken from abstracts from major computer science conferences, where each abstract is similar in length to a full manuscript and includes details satisfactory for critical appraisal. The search strategy was developed based on adjacent reviews studying AI in pediatric urology [7].

### Study selection

The eligibility criteria for studies were broad, to include all study types, where ML algorithms were used for clinical prediction. Models were included if they satisfied the following criteria:

1. The model is focused on a pediatric urology question (with statistics pertaining to a pediatric population)
2. The model includes patient outcomes or clinically relevant prognostic factors (e.g. only pathology prediction or segmentation/feature-engineering specific would be excluded)
3. The model uses ML algorithm for prediction (tree-based, support-vector machine (SVM), deep learning, gradient boosting, lasso regression, naïve Bayes, and ensemble methods. Traditional multivariable regression is not sufficient.

Two reviewers screened titles, abstracts, and full-texts. Discrepancy at each stage was adjudicated by a third reviewer. The reasons for exclusion at each stage were documented to ensure transparency and inform future research.

### Data extraction, analysis and model quality assessment

Two reviewers extracted data from each included article, and discrepancies were resolved by a third reviewer. The following details were collected: study details, patient characteristics, model objectives, model inputs/outputs, performance metrics, and clinical usability.

We qualitatively synthesized the collected data into tables, and assessed trends by year of publication, journal, topic of pediatric urology, and model characteristics. Meta-analysis was not performed within the context of this scoping review.

Each study was appraised with the APPRAISE-AI tool to determine the risk of bias for each included model [10,16]. This consisted of 24 methodological and reporting items reflecting best AI practices [16]. Two raters with ML expertise individually assessed each article for APPRAISE-AI score, and consensus was used for disagreements. Overall scores ranged from 0 to 100 points, with corresponding levels of study quality: scores from 0 to 19 indicate very low quality, 20 to 39 indicate low quality, 40 to 59 indicate moderate quality, 60 to 79 indicate high quality, and 80 to 100 indicate very high quality.

Spearman’s rank-order correlation was employed to assess the relationship between APPRAISE-AI study quality metrics and the year of publication. Since the APPRAISE-AI data did not follow a normal distribution, Spearman’s correlation was chosen as the appropriate non-parametric test. Spearman’s correlation coefficient ρ were reported with 95 % confidence intervals, calculated from bootstrapping 1000 samples. P-values <0.05 were considered statistically significant.

### Living scoping review and online repository

The scoping review will be updated annually in June, with repeated steps described above. The search will be modified as needed and will include articles that are submitted to the online repository (Supplementary Fig. 1). The models are then presented in the form of model cards (e.g. Supplementary Table 2). Model cards provide a snapshot of key elements (objective, AI method, data source, model input, model outcomes, measurements of predictive capability, and usability) of each model to allow ease of review by clinicians and researchers.

An online repository (www.aipeduro.com) hosting each of the AI models reported in this review will be updated every two months with submissions from clinical end-users. The criteria are the same as those for inclusion within this review. These will be incorporated into the scoping review on an annual basis, if not captured within the search of the living review.

A summary of recommendations based on the current synthesized literature and ciritcal appraisal was generated based on consensus agreement by the collaborative.

## Results

**Fig. 1:**
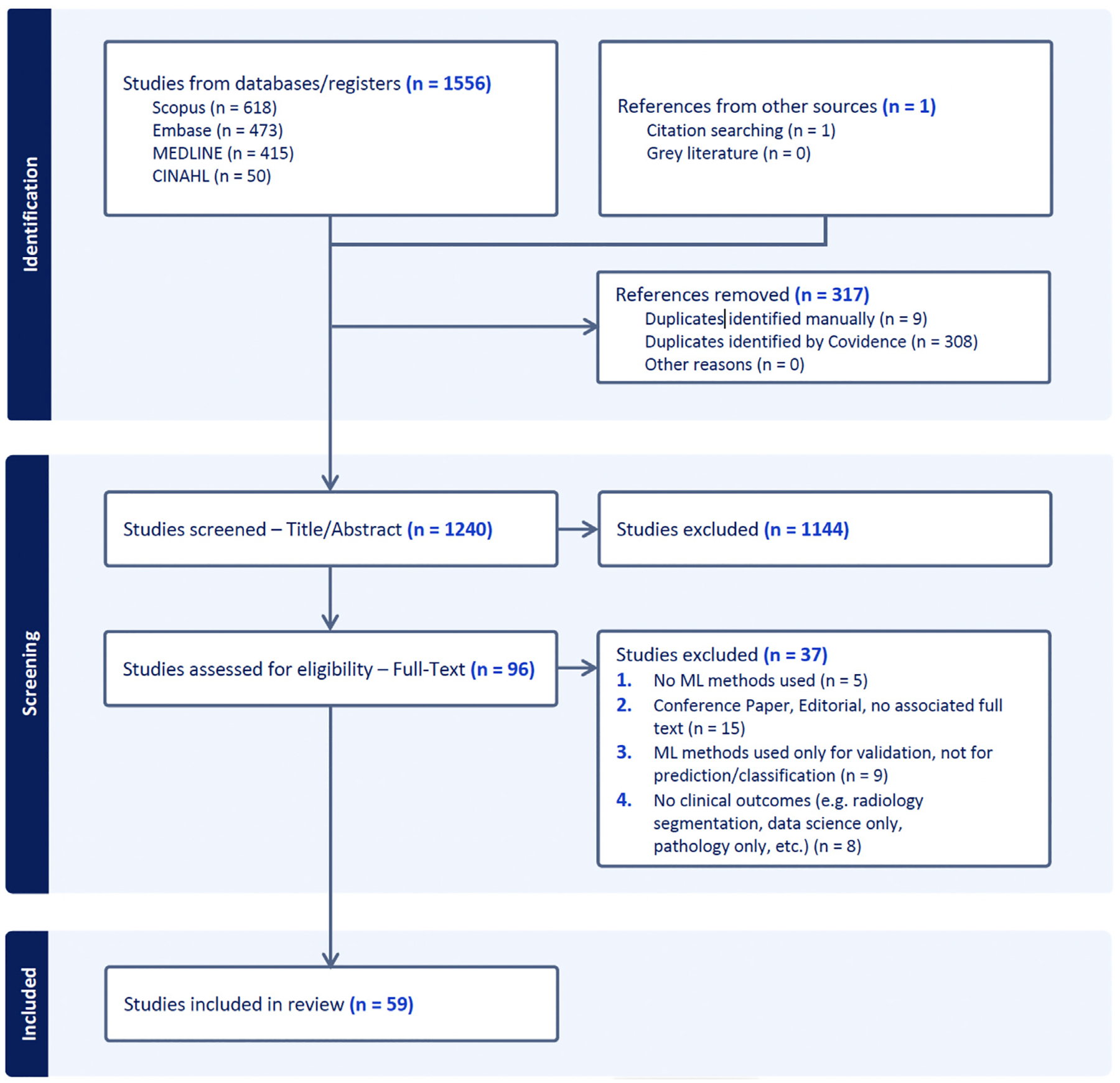
PRISMA flow chart of included studies.

A detailed overview of study identification, screening, and inclusion of studies is provided in the PRISMA flow chart (Fig. 1). From a total of 1557 records, we identified 59 studies that met inclusion criteria which were extracted for the following analysis.

### Study characteristics

**Fig. 2:**
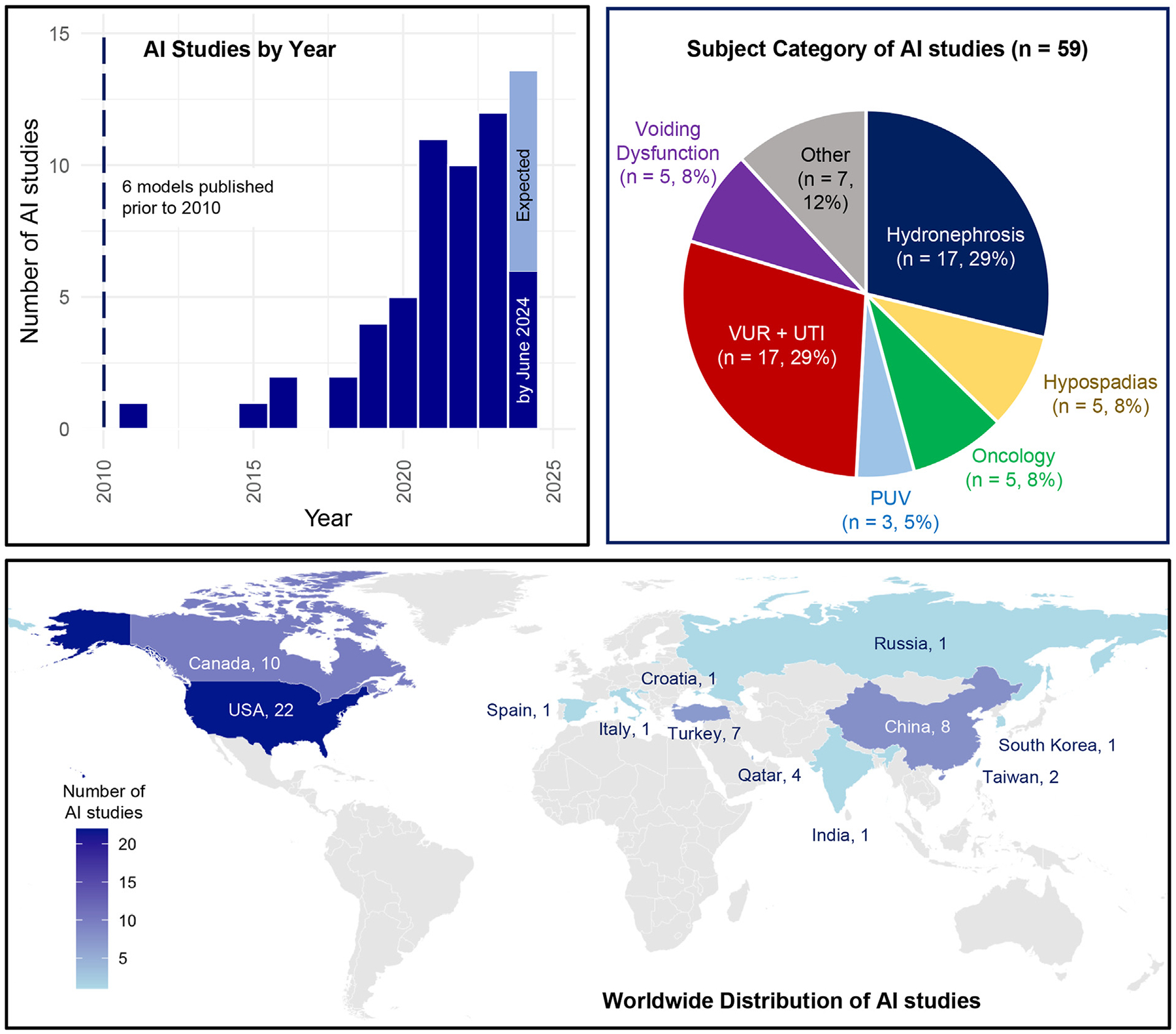
(A) Number of AI studies by year with cut-off at 2010, (B) study topic for each included study, and (C) Geographic distribution of published AI study by corresponding author institution.

Of the 59 published studies (Supplementary Table 3), 44 studies (75 %) were published after 2019, with the first study published in 1998 (Fig. 2a). The most common study topics were hydronephrosis and VUR/UTI providing 17 studies each (28 %), followed by hypospadias, oncology, and voiding dysfunction which each providing 5 studies (8 %) (Fig. 2b). The geographic distribution of published studies showed that USA (22 studies, 37 %), Canada (10 studies, 17 %), China (8 studies, 14 %), and Turkey (7 studies, 12 %) published the highest number of studies (Fig. 2c). The studies were retrieved from 30 unique peer-reviewed journals, with Journal of Urology (10 studies, 17 %), Journal of Pediatric Urology (9 studies, 15 %), and computer science conferences (5 studies, 8 %) being the most frequent.

Of the 59 published studies, 17 (29 %) used multiple ML algorithms. The most employed algorithms were neural networks (35 studies, 59 %), SVM (21 studies, 36 %), and tree-based models (19, 32 %). The primary model objective was prediction (27 studies, 46 %), classification (23 studies, 39 %), automation (6 studies, 10 %), and recognition (3 studies, 5 %). Of these models, a useable coding repository, web application, or dataset is provided in 14 studies (24 %).

### Model quality assessment and appraisal

**Fig. 3:**
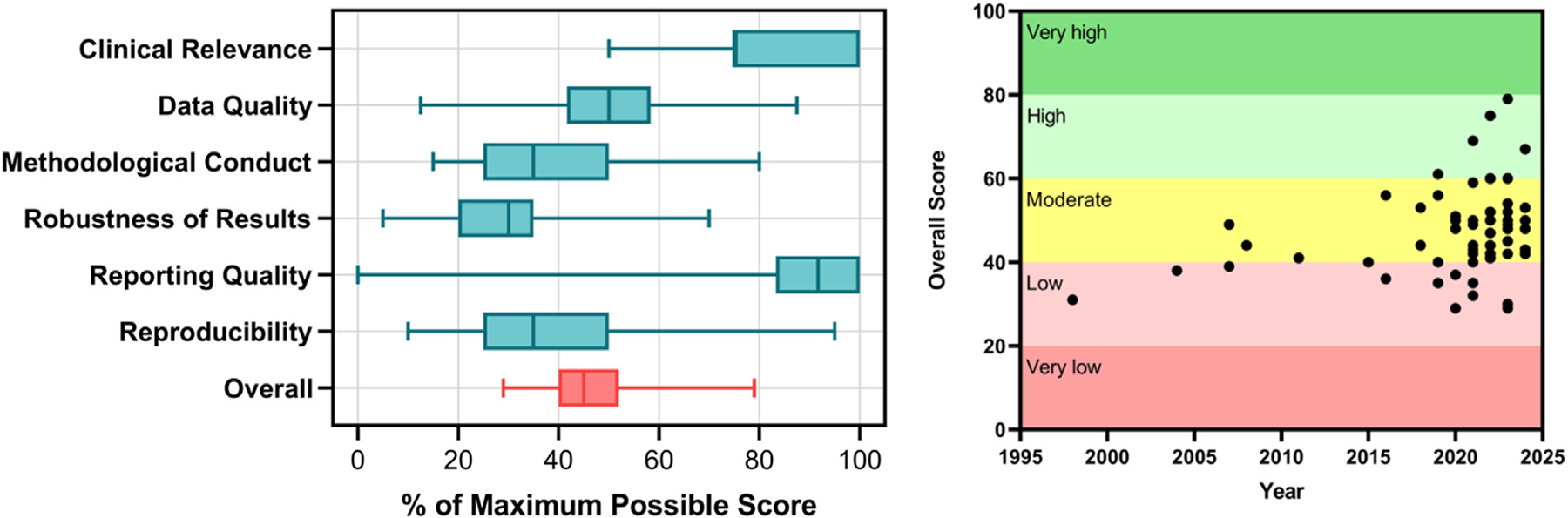
(Left) Model quality by each APPRAISE-AI domain and (Right) overall model quality over time.

APPRAISE-AI was applied to all included studies to assess model quality (Fig. 3). Median overall score was 47 (moderate quality) and ranged from 29 (low quality) to 79 (high quality). In total, 12 studies (20 %) were low quality, 39 studies (66 %) were moderate quality, and 8 studies (14 %) were high quality (1 hydronephrosis, 1 hypospadias, 1 PUV, 4 VUR/UTI, and 1 voiding dysfunction) (Supplementary Table 3). The two strongest APPRAISE-AI domains were clinical relevance and reporting quality, while the three weakest were methodological conduct, robustness of results, and reproducibility. The most missed areas were adequate description of data sources (87 %), sample size calculation (95 %), error analysis (88 %), and transparency (85 %) (Supplementary Fig. 2). Notably, the robustness of results category on quality assessment was poorly reported across the field. Overall, study quality has improved over time indicated by a regression coefficient of 0.27 (95%CI of 0.02, 0.50, p = 0.03). Among the 31 studies published in 2021 and prior, only 1 was high quality (3 %), while 7 of the 28 studies (25 %) published after 2021 were high quality (p = 0.04).

## Discussion

AI models are being developed across pediatric urology and will soon influence care. This scoping review showed a wide variety of AI models in pediatric urology targeted towards hydronephrosis, VUR and UTI, hypospadias, oncology, and voiding dysfunction. Since 2018, there has been an exponential growth in the number of studies, and most current studies originate from North America and Asia. These models aim to personalize treatment decisions, improve prognostication, and standardize subjective criteria. The quality of models is improving based on quality assessment, and most studies at this time were of moderate quality.

Three models warrant additional mention for their use of clinical trial data that is amenable for translation [17], high model quality [5], and a novel approach to standardizing a long-standing problem [3]. Using data from RIVUR and CUTIE patients, Bertsimas created a model which provided decision trees for which patients benefit from an invasive VCUG after their first presentation for a febrile UTI [17]. This approach employed ML to show and quantify which at-risk groups (younger, female, concurrent voiding dysfunction, etc.) were most likely to benefit to aid in clinical decision-making for a common problem faced by pediatric urologists. Next, routine renal bladder ultrasound parameters were shown to predict which patients with prenatal hydronephrosis could safely defer a diuretic renogram by Khondker et al. [5]. While the model showed modest performance, it was externally validated, code was openly shared, and web-application was freely useable, and reporting was robust warranting a high model quality score [5]. Lastly, ML was used to standardize classification of hypospadias phenotypes which offered a new approach to multi-decade issue [3]. The solution was elegant due to its inclusion of multiple raters to assess distal vs. proximal hypospadias so that the model learned to predict the consensus and remained more reliable than individual raters.

We used APPRAISE-AI to assess and summarize methodological rigor and reporting quality. Among the included studies, 14 % were of high quality. Studies consistently assessed clinically relevant problems and adequately appraised their work. However, there were significant concerns regarding data quality, methodology, robustness, and reproducibility which often limited model quality. This is similar to other fields of urology and especially true amongst pediatric hydronephrosis models [16,18]. Reassuringly, there is evidence that model quality has been improving over time. With greater adoption of reporting guidelines, such as TRIPOD-AI, STARD, STREAM-URO, and PRISMA-AI [10,19–22] we expect this improvement to continue in future research. Based on the themes within this literature, we highlight specific recommendations for AI studies in pediatric urology in Table 1.

A living review provides an opportunity for ongoing synthesis of this rapidly advancing topic. While the review is planned for annual updates, the online repository will be updated frequently and hopefully be a useful resource for pediatric urologists, data scientists, among others. Over time, iterative changes will be responsive to updated literature and needs from clinical end-users.

### Limitations

There are limitations to this scoping review and online repository. First, although we included 59 studies, we did not perform a systematic review or meta-analysis of included outcomes which limits our ability to synthesize outcomes. We believe this is best served with topic-focused reviews on specific topics, such as hydronephrosis or VUR. Next, we used APPRAISE-AI to assess model quality which was published after the publication date of included studies and best practices in AI have changed over time. However, APPRAISE-AI is well-aligned with established reporting guidelines such as the TRIPOD statement with the ability to express quality over the field [19,23]. Last, a living scoping review and online repository will provide a reference of AI models but will rely on regular updates from the collaborative team to remain updated, and adaption over time to the needs of those using these models.

## Conclusion

This scoping review and online repository (www.aipeduro.com) establishes a comprehensive synthesis of the current state of AI in pediatric urology. Models have been applied to multiple conditions, and despite most studies being moderate quality, there is gradual improvement over time. We hope that AI-PEDURO will foster greater understanding of AI models, support external validation, improve implementation of these AI models in clinical care and facilitate the recommendations suggested in this work, while adapting over time.

## Supplementary Material

Supplemental mmc2

Supplemental mmc1

## Figures and Tables

**Table 1:** Summary of recommendations to improve AI studies in pediatric urology, examples provided for pediatric hydronephrosis.

Areas for Improvement	Recommendations
**Data quality**	Adequately describe the patients included and variables, provide a data dictionary where feasible Include patients from multiple institutions, when possible Provide description of the standard of care and possible institutional variation from this that may impact outcome Provide a sample size calculation
**Outcome definitions**	Use variables and outcome definitions that are standard for the topic [e.g. SFU grade, anteroposterior renal diameter, etc.] When using subjective outcomes [e.g. indication for surgery], provide associated objective outcomes [e.g. ultrasound or renogram parameters]
**Methodology**	Clearly describe data pre-processing, model development, and tuning of hyperparameters Ensure the cohort(s) are separated into training/testing cohorts Incorporate validation methods to avoid overfitting (i.e. bootstrapping, internal cross-validation, external validation)
**Evaluation**	Compare AI models with available non-AI methodology (logistic regression), and previous AI models (if available) Evaluate AI models based on discrimination, calibration, net benefit, and bias assessment
**Reproducibility**	Share models, code, and data in available repositories (i.e. GitHub) Make models accessible via web applications (i.e. RShiny, streamlit), nomograms, or useable outputs (i.e. trees, regression weights, etc.)
**Clinicians and reviewers**	Invite co-authors and reviewers with expertise in AI to assist and evaluate technical aspects of AI studies Perform literature review to familiarize with current AI models that have been applied to current research problem Utilize appropriate reporting guidelines, such as STREAM-URO or TRIPOD-AI

## Appendix A. Supplementary data

Supplementary data to this article can be found online at https://doi.org/10.1016/j.jpurol.2025.01.035.

## Abbreviations

AI: Artificial intelligence
AIPEDURO: Artificial Intelligence in PEDiatric UROlogy
ANN: Artificial neural network
CNN: Convolutional neural network
ML: Machine learning
SVM: Support vector machine
UTI: Urinary tract infection
VUR: Vesicoureteral Reflux
