# Supplementary material for: A living scoping review and online repository of artificial intelligence models in pediatric urology: Results from the AI-PEDURO collaborative
Source: J Pediatr Urol. Author manuscript; Available in PMC 2026 Feb 16. (PMC12908176; doi:10.1016/j.jpurol.2025.01.035)
Supplement: Supplemental mmc2 [file NIHMS2144933-supplement-Supplemental_mmc2.docx]

**Supplementary Table 3**: Description of included AI models in pediatric urology

| **Study** | **Objective** | **AI Approach** | **Data Source(s)** | **Model Input Variables** | **Model Outcome and Performance**  **[Validation approach]** | **Usability and Data Availability** | **Model Quality (APPRAISE-AI)** |
| --- | --- | --- | --- | --- | --- | --- | --- |
| **Hydronephrosis and Pyeloplasty** | | | | | | | |
| Bagli 1998 [1] | To predict sonographic outcome after pyeloplasty in children with UPJO | ANN | Institutional series (100 children) | 242 clinical variables | Predicting sonographic outcomes after pyeloplasty: AUC of 1.0 and accuracy of 1.0 [on 16 testing set]    [Holdout validation] | No available code or dataset, no usable predictive tool | Low |
| Blum 2018 [2] | To predict clinically significant hydronephrosis caused by UPJO | SVM | Institutional series (55 children) | 45 clinical factors including:  drainage curve features (skewness, kurtosis), drainage half-time, concentration at 30 min | Clinically significant hydronephrosis  AUC of 0.96, accuracy of 93%    [Leave-one-out analysis] | No available code or dataset, no usable application | Moderate |
| Cerrolaza 2016 [3] | To define sonographic markers for hydronephrotic kidneys that predict need for diuretic nuclear renography | SVM | Institutional series (50 children) | 131 parameters from 2D US: Size (size of collecting system, renal parenchyma)  Geographic shape (circularity ratio eccentricity),  Curvature descriptors (local curvature) | T1/2 > 20 mins: AUC of 0.98, accuracy of 0.96  T1/2 > 30 mins: AUC of 0.94, accuracy of 0.78  T1/2 > 40 mins: AUC of 0.94, accuracy of 0.78    [Leave-one-out analysis] | No available code or dataset, no usable application | Moderate |
| Dhindsa 2018 [4] | To classify the severity of prenatal hydronephrosis from renal US images. | CNN | Institutional series (773 patients, 2492 US images) | 256 x 256 pixel images of renal US | Classifying prenatal hydronephrosis:  All grades: accuracy: 51.5%; F1: 0.52; High v. low risk hydronephrosis: accuracy of 79.8%; F1: 0.80  *Also available vs. SFU grades and vs. controls*  [Cross validation] | No available code or dataset, no usable application | Moderate |
| Drysdale 2021[5] | To predict risk of and time-to re-intervention after pyeloplasty | Logistic Lasso | Institutional series (543 patients) | 43 clinical factors, most importantly: anteroposterior diameter on US | Risk of re-intervention: AUC of 0.86    Time to re-intervention: c-index of 0.78    [Leave-one-out analysis] | Freely-available web application, publicly available code repository | Moderate |
| Erdman 2020 [6] | To predict obstructive hydronephrosis requiring surgery from renal US in children with prenatal hydronephrosis | CNN | Institutional series (294 patients, 1645 US images) | 256 x 256 pixel images of renal US | Requiring surgery: AUC of 0.93, and accuracy of 0.58    [Holdout validation] | No available code or dataset, no accessible predictive tool. Model explainability provided with Grad-CAM (overlayed on input images). | Moderate |
| Guan 2022 [7] | To diagnose and grade UPJO from US images | Segmentation and CNN | Institutional series (229 patients, 3289 US images) | 810 x 608 pixels transverse and coronal section renal US images | Diagnose and grade UPJO: accuracy: 92%  AUC grade 0-2: 0.98  AUC grade 3: 0.90  AUC grade 4: 0.97  [Holdout validation] | No available code or dataset. | Moderate |
| Khondker 2023 [8] | To sensitively predict the risk of renal obstruction on diuretic renography using routine reported US findings | RF | Institutional series (304 patients, external validation 64 patients) | Age, sex, laterality, kidney length, APD, SFU grade on US report | T1/2 > 20 min: AUC of 0.76-0.84  [Cross validation and external validation] | Available clinical model provided, code repository provided | High |
| Lorenzo 2019 [9] | To predict need for surgical intervention in prenatal hydronephrosis | Boosted decision tree, neural network | Institutional series (557 children) | Age, gender, affected side, SFU grade, renogram findings, ureteral dilatation, anteroposterior diameter | Surgical intervention: AUC of 0.90, accuracy of 0.87    [Holdout validation] | No available code or dataset, no usable application. Linked to the Microsoft Azure platform. | Low |
| Ostrowski 2023 [10] | To classify hydronephrosis on renal US imaging according to the SFU system | CNN | Institutional series (710 children, 4659 US images) | Sagittal and transverse renal images | SFU grade 0-IV: overall accuracy of 0.82 and within one grade accuracy of 0.98  [Cross validation] | No available code or usable application. | Moderate |
| Roshanitabrizi 2021 [11] | To automate the assessment of obstruction severity in pediatric hydronephrosis | CNN | Institutional series (49 patients) | 128x128 pixel renal US images | Prediction of obstruction severity (severe vs. non-severe hydronephrosis). Accuracy: 0.83  [Cross validation] | No available code or dataset. | Moderate |
| Sloan 2023 [12] | To identify high-grade hydronephrosis from renal US extracted features | SVM | Institutional series (90 children, 592 US images) | Radiomic features from renal US including contrast, correlation, difference entropy/energy, and gray-level co-occurrence matrix | High (SFU III-IV) vs. Low grade (SFU I-II) hydronephrosis: AUC of 0.86, sensitivity of 0.76, specificity of 0.88  [Cross validation] | No available code or dataset, no usable application. | Moderate |
| Seckiner 2011 [13] | To predict management (observation vs. surgery) for UPJO | ANN | Institutional series (53 children) | Age, sex, renal pelvic diameter, laterality, split renal function on radionuclide scan, presence of renal scar on DMSA scan, urine culture results, presence of symptomatic urinary infections | Prediction of the need for surgery versus observation:  Training: sensitivity - 92%, specificity - 77%  Test group: sensitivity - 75%.  [Cross validation] | No available code or dataset, no usable application. | Moderate |
| Smail 2020 [14] | To predict SFU grade of hydronephrosis | CNN;  Layer-wise propagation to visualize output. | Institutional series (2420 sagittal hydronephrosis US images) | 256 x 256 pixel images of sagittal US | SFU grade 0-IV: F1 score of 0.49, accuracy of 0.51  Mild vs. severe: F1 score of 0.78, accuracy of 0.78  SFU II vs. SFU III: F1 score of 0.71, accuracy of 0.71  [Cross validation] | Layer-wise propagation to visualize output. Datasets available on request. No available code or predictive tool. | Moderate |
| Tabrizi 2021  [15] | To predict severe ureteropelvic junction obstruction from renal US images | CNN | Institutional series (54 US images) | Renal US, coronal image | Predicting obstruction as T1/2> 20 mins: accuracy of 0.78, sensitivity of 0.62, specificity of 0.83  [Holdout and cross validation] | No available code or dataset, no usable application. | Low |
| Tsai 2022  [16] | To evaluate the diagnostic performance of deep learning techniques in classifying kidney images as normal or abnormal. | CNN with transfer learning | Institutional series (1599 images) | Renal US images (224x224 pixels) | Overall: Accuracy 92.9%, AUC 0.96  *Also available, stones, cysts, hyperechogenicity, space-occupying lesions, hydronephrosis.*  [Cross validation] | No available code or dataset, no usable application. | Moderate |
| Weaver 2023a [17] | To analyze MAG3 renal scans using machine learning to predict renal complications in prenatal hydronephrosis | RF, CNN | Institutional series (152 children) | Deep-learning: Radiotracer concentration on MAG3 study images, over time  RF: Age, sex, race, laterality, differential function measurements, T1/2 | Renal complications (defined as decline of greater than 5% in renal function of the affected kidney, increased parenchymal thinning on US, worsening hydronephrosis, new onset flank pain, or occurrence of pyelonephritis)  CNN: AUC of 0.78, accuracy of 0.73  RF: AUC of 0.67, accuracy of 0.62  [Holdout and cross validation] | No available code or dataset, no usable application. | Moderate |
| **Hypospadias and Penile Curvature** | | | | | | | |
| Abbas 2022 [18] | To capture automated measurements of penile curvature based on 2-dimensional images | CNN | Nine 3D-printed penile models (900 image dataset) | Image of penile model captured from smartphone | Cropping penile area: mean average precision of 0.99  Segmenting shaft: dice similarity coefficient 0.98  Curvature angle estimation: mean absolute error 8.5 degrees  [Cross validation] | No available code or usable application. Open dataset is provided. | High |
| Abbas 2023a [19] | To automate the calculation of the POST score in hypospadias using patient images alone. | CNN | Institutional hypospadias image database (691-image dataset) | Image of hypospadias with landmarks including distal midline mucocutaneous junction, glanular knobs, and glanular-coronal junction | Localizing glans area: mean average precision of 0.99, sensitivity of 0.99  Predicting POST landmarks: NME 0.072, MSE 0.001, 0.025 failure rate  [Cross validation] | Open website was developed where clinicians can register to upload images and test the model. No code or dataset is provided. | Moderate |
| Baray 2023 [20] | To automate penile curvature measurement using 2 points without requiring arc area. | CNN | Nine 3D-printed penile models (913 image dataset); 4 intraoperative penile images from web scraping | Image of penile model or intraoperative image of penis captured from smartphone | Penile area localization: mAP0.5 of 0.99  Angle estimation from model images: mean absolute error of 4.5 degrees  [Cross validation] | Dataset available upon-request. No code or usable application is provided. | Moderate |
| Fernandez 2021 [21] | To classify distal versus proximal hypospadias | CNN | Hypospadias image database (1169 anonymized images) | Image of hypospadias | Distal vs. proximal hypospadias: accuracy 90%  [Holdout validation] | No available code or dataset, no usable application | Moderate |
| He 2024 [22] | To identify hypospadias classification from genital anthropometric features | RF  and SVM | Institutional series (259 children); prospectively collected institutional testing set (130 children) | Penoscrotal distance, anogenital distance, 2D:4D finger ratio | Based on the prospectively collected testing set 3-classification models.  Distal: sensitivity of 12% (RF), 0% (SVM)  Midshaft: sensitivity of 57% (RF), 77% (SVM)  Proximal: sensitivity of 65% (RF), 53% (SVM)  [Holdout validation] | No available code or dataset, no usable application | Moderate |
| **Oncology** | | | | | | | |
| Li 2023 [23] | To predict the risk of chemotherapy-induced myelosuppression (CIM) in children with Wilms’ tumor. | Extreme Gradient Boosting, LR, RF, Lasso, SVM, CatBoost | Institutional series (437 children, 1433 chemotherapy cycles) | Age, gender, height, weight, tumor stage, COG grade, the routine hematologic index and  biochemical index, routine urinalysis, the type of chemotherapy drugs used, chemotherapy  cycles | To predict grade ≥2 CIM: AUC of 0.981 in the training set, AUC of 0.90 in the test set, sensitivity 76%, specificity 93%.  [Holdout and cross validation] | Data available upon request. No code or predictive tool provided. | Moderate |
| Ma 2022 [24] | To preoperatively predict stages (stage I and non-stage I) Wilms tumor in pediatric patients | SVM | Institutional series (118 patients) | Pre-op portal venous-phase CT images (15 features: 2 first-order features, 13 texture features) | To predict WT staging: training set: AUC of 0.79, accuracy of 75%; test set: AUC of 0.81, accuracy of 79%  [Holdout and cross validation] | Dataset and code available as supplementary material upon-request. | Moderate |
| Sharaby 2023 [25] | To assess the response of Wilms' tumors to preoperative chemotherapy | SVM | Institutional series (63 pediatric patients) | Regions of interest from CT images (incl. Shape features, functionality-based features, appearance features) | Prediction of tumor response to neoadjuvant chemotherapy: Accuracy of 95%, Sensitivity of 96% Specificity of 94%, F1-score of 0.97  [Cross validation and leave-one-analysis] | Data available on request. No available code or dataset. | Low |
| Song 2024 [26] | To classify CT images for identification of Wilms and non-Wilms tumors. | SVM, RF, LDA, Adaboost, Gaussian process, Auto-Encoder, LR-Lasso, Decision tree, Naïve Bayes. | Institutional series (82 children) | CT images (texture features) and clinical features (age, sex) | Corticomedullary phase:  Validation set highest AUC: 0.79  Training Set AUC: 0.81  Testing Set AUC: 0.43  Nephrogenic phase:  Validation set highest AUC: 0.66  Training Set AUC: 0.78  Testing Set AUC: 0.69  [Cross validation] | Dataset available upon request to authors. No available code or tool. | Moderate |
| Zhu 2023 [27] | To discriminate Wilms tumor (WT) from non-WTs | CNN | Institutional series (364 patients) | 224 x 224 regions of interest from CT images | Discriminating Wilms Tumor: AUC: 0.83; accuracy: 0.78, sensitivity: 0.56, specificity: 0.84  [Holdout validation] | Dataset available upon request to authors. No available code or tool. | Moderate |
| **Posterior Urethral Valve** | | | | | | | |
| Abdovic 2019 [28] | To predict late-presenting PUV in boys with urinary symptoms | ANN | Institutional series (201 uroflows) | Age, max flow-rate, time to peak flow, volume, voiding time, flow time, average flow rate | Late-presenting PUV: AUC 0.98, and accuracy of 0.93    [Cross validation] | Freely-available web application, publicly available code repository | Moderate |
| Kwong 2022 [29] | To predict risk of chronic kidney disease (CKD) progression, need for renal replacement therapy (RRT), and clean-intermittent catheterization (CIC) | Random survival forest | Institutional series (103 patients),  one external institutional series (22 patients) | Nadir Creatinine, one-year eGFR, VUR grade on VCUG, and US findings of renal dysplasia | CKD Progression: c-index of 0.77  RRT: c-index of 0.95  CIC: c-index of 0.70    [Holdout validation] | Freely-available web application, publicly available code repository, no dataset. | High |
| Yin 2020 [30] | To diagnose children with PUV | CNN with transfer learning | Institutional series (157 children: 3504 sagittal US, 2558 transverse US) | Sagittal and/or transverse features of renal US | PUV, with multiple views: AUC of 0.96 and accuracy of 0.93    [Cross validation] | Publicly available code repository, no available tool | Moderate |
| **Vesicoureteral Reflux and Urinary Tract Infections** | | | | | | | |
| Arlen 2016 [31] | To predict the probability of breakthrough fUTI in children with primary VUR. | 2-hidden node neural network | Institutional series (384 VCUGs) | Age, gender, laterality, percentage PBC at VUR onset, VUR grade right/left, VUR onset right/left (filling or voiding), complete ureteral duplication, number of UTIs prior to VUR diagnosis (2 vs. <2), history of fUTI, and history of BBD. | Predict breakthrough febrile UTI occurrence:  AUC 0.76  [Holdout validation] | No publicly available code or dataset. Authors state that a web-based publicly available model is in production. | Low |
| Bertsimas 2021 [32] | To predict which patients with VUR are most likely to benefit from continuous antibiotic prophylaxis | Optimal classification trees | Multi-institutional trial dataset (RIVUR, 607 patients) | Race, gender, VUR grade, serum creatinine, prior UTI symptoms, weight percentiles | Risk of recurrent UTI: AUC of 0.82    [Holdout validation] | Easily accessible decision trees and available mobile application | Moderate |
| Dubrov 2021 [33] | To predict the outcomes of a single endoscopic injection of DxHA for correction of VUR | Multilayer ANN (multilayer perceptron) with two hidden layers and a sigmoid function of neuronal activation | Multi-institutional series (582 children, 783 ureteric units operated on) | VUR grade, age, sex, presence of ureteral duplication, ureteral dilatation index | To predict outcome of DxHA injection treatment: AUC of 0.77, correct prognosis 74.5%, sensitivity 85.5%, specificity 65.3%  Clinic 1: AUC of 0.72, correct prognosis 69%, sensitivity 91%, specificity 30.2%  Clinic 2: AUC of 0.8, correct prognosis 78%, sensitivity 70%, specificity 82%  [Holdout split] | No available code or dataset, no usable predictive tool. | Moderate |
| Eroglu 2021 [34] | To determine VUR grade using images from VCUGs | Hybrid CNN (+ K-nearest neighbors or + SVM) | Institutional series (1228 images) | Raw VCUG images | To predict each normal and each VUR grade: AUC of 0.99, and accuracy of 97%    [Holdout validation] | No available code or dataset, no usable predictive tool | Low |
| Ganapathy 2023 [35] | To predict renal scarring in pediatric population with VUR | Logistic Regression, Discriminant Analysis, Bayesian Logistic Regression, Naïve Bayes, Decision Tree | Institutional series (94 children) | Kidney injury molecule-1 (KIM-1), Neutrophil gelatinase–associated lipocalin (NGAL), Urinary creatinine, Ratios of NGAL and KIM-1 to urinary creatinine | To predict the presence of renal scarring:  Logistic Regression: AUC = 0.83  Discriminant Analysis: AUC = 0.83  Bayesian Logistic Regression: AUC = 0.83  Naïve Bayes: AUC = 0.81  Decision Tree (C5.0): AUC = 0.83  [Unspecified validation] | No available code or dataset, no usable predictive tool | Low |
| Kabir 2024 [36] | To determine VUR severity using quantitative features extracted from VCUG image | 6 classification algorithms (MLP, Tree-based, SVM, etc.) | Online image scraping (113 VCUG images) | VCUG extracted features: max ureter width, ureter tortuosity, UPJ width, UVJ width, ureter curvature, ureter area, pelvicalyceal outline length, pelvicalyceal region area, pelvicalyceal outline curvature | Assessing binary VUR grade (low [I-III] vs. high [IV-V]).  MLP: AUC of 0.96, F1-score of 0.91  SVM: AUC of 0.95, F1-score of 0.90  [leave-one-out analysis] | No available code or usable tool. Web-scraped dataset is provided. | Moderate |
| Keskinoglu 2020 [37] | To determine a diagnosis of VUR versus UTI | ANN | Institutional series (611 children) | 39 variables (clinical, laboratory, and US findings) | VUR/UTI: AUC of 0.81, and precision of 0.78  [cross validation] | No available code or dataset, no usable predictive tool | Low |
| Khondker 2021 [38] | To predict high-grade VUR from quantitative features annotated from VCUGs | RF | Web scraping (41 renal units), institutional series (44 renal units) | Ureter tortuosity, UPJ width, UVJ width, and maximum ureter width on VCUG | High-grade VUR: AUC of 0.83, accuracy of 0.90    [Leave-one-out analysis] | Freely-available web application, publicly available dataset. | High |
| Khondker 2022 [39] | To predict VUR grade from quantitative features annotated from VCUGs | RF | Multi-institutional series (1248 renal units, from 4 institutions, 1 web-scraped dataset, 1 community dataset) | Ureter tortuosity, proximal ureter width, distal ureter width, and maximum ureter width on VCUG | Grade II vs III vs IV vs V: AUC of 0.75-0.94 (training), AUC of 0.72-0.91 (external).  [Cross validation and external validation] | Freely-available web application, online code repository, performed bias assessment. Available reliability in outcome. freely available web-scraped database but no patient databases. | High |
| Knudson 2007 [40] | To predict the chance of early VUR resolution. | Linear SVM | Institutional Series (205 children) | Age, gender, presenting symptoms, reflux grade, laterality, whether reflex occurred during filling or voiding, initial bladder volume at onset, presence of complete ureteral duplication | Prediction of resolution 1 year after diagnosis: AUC of 0.819.  Prediction of resolution 2 years after diagnosis: AUC of 0.86  [Holdout validation] | Model/Prognostic Calculator is readily available on the internet through JavaScript. | Low |
| Li 2024 [41] | To develop and validate a deep-VCUG with ensemble learning for automatic VUR grading from VCUG images | CNN, Ensemble learning | Multi-institutional series (1948 images, from 5 institutions) | 512 x 512 pixels VCUG images | Unilateral VUR: AUC of 0.96 (internal), AUC of 0.94 (external); Bilateral VUR: AUC of 0.96 (internal), 0.92 (external)  [split-sample validation, external validation] | Code is publicly available. Dataset is available upon-request. No usable tool. | High |
| Logvinenko 2015 [42] | To predict VUR grade from renal and bladder US findings on the same day | ANN | Institutional series (2259 children) | RBUS findings, sex, age, circumcision status (in boys), febrile UTI, first (vs. recurrent) UTI | Any VUR: AUC of 0.69  VUR grade > II: AUC of 0.67  VUR grade > III: AUC of 0.79    [Unclear validation method] | No available code or publicly available tool. | Moderate |
| Seckiner 2008 [43] | To predict the resolution of VUR | ANN | Institutional series (145 ureteric units) | Age, sex, the cause and grade of VUR, the affected ureter, the type of treatment, existence  of renal scar on DMSA scan, follow-up times, the number of  injection | VUR resolution: accuracy of 0.98  VUR improvement: accuracy of 1.0  VUR persistent or worse: accuracy of 0.92    [Holdout validation] | No available code or dataset, no usable predictive tool. | Moderate |
| Serrano-Durba 2004 [44] | To predict the results of endoscopic treatment for VUR | ANN | Institutional series (261 ureteric units) | Age, sex, cause/grade of VUR, type/number of implanted substance, number of treatments, affected ureter, endoscopic findings, type of cystography | Success of endoscopic treatment: AUC of 0.77    [Holdout validation] | Full code is provided, no usable predictive tool. | Low |
| Estrada 2019 [45] | To predict the probability of recurrent UTI and associated VUR after initial UTI but before VCUG | Optimal classification Trees, RF, gradient-boosted trees | Multi-institutional trial dataset (RIVUR, 305 patients; CUTIE, 195 patients) | Age, gender, race, weight, antibiotic resistance in urine culture, urine protein, dysuria, medications, antibiotics in last 6 months, blood pressure | VUR-associated recurrent UTI: AUC of 0.76    [Holdout and cross validation] | Easily accessible decision trees, freely accessible GitHub and available mobile application | High |
| Lee 2022 [46] | To predict the recurrence of UTI after ^99m^Tc-DMSA renal scan | CNN | Institutional series (180 patients) | Pre-processed ^99m^Tc-DMSA images | Recurrent UTI: accuracy of 0.91    [Cross validation] | No available code or dataset, no usable predictive tool | Moderate |
| Wang 2024 [47] | To reliably predict dilating VUR from early postnatal US in patients with hydronephrosis | Optimal Classification Tree (OCT) | Institutional series (280 patients, 530 renal units) | Patient demographics (age at US, sex, kidney laterality) and UTD features (primary antero-posterior diameter, central calyceal dilation, peripheral calyceal dilation, parenchymal thickness, parenchymal appearance, distal ureteral dilation) | Dilating (>Gr3) VUR: AUC of 0.81 (95% CI: 0.73-0.88)  [Holdout and cross validation] | Julia language was used. Dataset not available. Final prediction model uses a tree structure that is available for clinical use. | Moderate |
| **Voiding Dysfunction and Detrusor Overactivity** | | | | | | | |
| Ge 2022 [48] | To classify and predict bladder compliance from a novel method that measures intravesical pressure during the VCUG examination without extra UDS. | SVM, RF, LR, Perceptron, XGBoost, and Naive  Bayes | Institutional series (52 patients) | Time-domain and wavelet features extracted from the bladder pressure measurement, sex, max pressure of bladder before urination, max perfusion | Differentiation of abnormal and normal bladder compliance:  SVM (RBF Kernel): AUC 0.87, Sensitivity 70.4%, Specificity 84.5%, Accuracy 79.1%  [Cross validation] | No available code, dataset, or usable application. | Moderate |
| Hobbs 2022 [49] | To identify detrusor overactivity from urodynamic studies in the spina bifida population | SVM, Dimensionality reduction | Institutional series (805 urodynamic studies) | 15 features from urodynamic study (time-based and frequency-based), after principal component analysis | Time-based detrusor overactivity: AUC of 0.92    Frequency-based detrusor overactivity: AUC of 0.91    [Holdout validation] | Influence of important predictors provided, no available data, or predictive tools provided. | Moderate |
| Yuan 2024 [50] | To develop an efficient bladder compliance screen approach before UDS | CNN | Single institutional series (UDS: 301 cases, perfusion: 52 datasets) | Bladder compression curve data: 624 samples of length 500 from the perfusion dataset and 3993 samples of length 500 from the UDS dataset | UDS dataset: Accuracy = 83%, AUC = 0.89, F1 = 0.84  Perfusion dataset: Accuracy = 79%, AUC = 0.85, F1 = 0.79  Mixed dataset: Accuracy = 79%, AUC = 0.85, F1 = 0.79  [Cross validation] | No available code or dataset. | Moderate |
| Wang 2021a [51] | To identify detrusor overactivity from urodynamic studies | Manifold learning | Institutional series (799 urodynamic studies) | Demographics, raw tracings of vesical  pressure, abdominal pressure, detrusor pressure, infused volume, annotations | Detrusor overactivity: AUC of 0.84    [Cross validation] | Extensive description of model development and performance, no available code | Moderate |
| Weaver 2023b [52] | To develop deep  learning models of VUDS to categorize severity of bladder dysfunction in patients with spina bifida | Individual and ensemble model (RF, CNN from tracing and imaging, respectively) | Institutional series (306 VUDS from 256 patients) | *Clinical*: Age, Pressure at 25, 50, 75% EBC; presence of VUR, leak, bladder shape, detrusor sphincter dyssynergia, post-void residual, %EBC achieved  *Urodynamics*: Tracings at 25, 50, 75% EBC; detrusor pressures  *VUDS*: Fluoroscopic images | *Ensemble model,* accuracy of 75%  No/mild: AUC of 0.75-0.85; Moderate dysfunction: AUC of 0.64-0.75;  Severe dysfunction: AUC of 0.82-0.89  [Cross validation] | Multiple model performances provided for each sub-model, reliability of ground truth measured, no available usable model. | High |
| **Miscellaneous** | | | | | | | |
| Celik 2020 [53] | To predict renal scarring in children with lower urinary tract dysfunction | 7 classification algorithms (XGBoost, ANN, SVM, etc.) | Institutional series (75 children), SMOTE re-sampling of original dataset (106 cases) | 18 features from clinical findings, US, renography, and VUDS, including bladder trabeculation, bladder wall thickness, presence of VUR, DRF, UTI episodes, etc. | Renal scarring defined as subsequent DRF < 40 or visible scarring/atrophy on renography.  [Cross validation]  Original database favored XGBoost: AUC of 0.83, accuracy of 0.91  [Holdout and cross validation] | No available code or dataset, no usable application. | Low |
| Eksi 2022 [54] | To predict the likelihood of orchiectomy rather than orchiopexy in testicular torsion using preoperative features | RF | Institutional series (256 children in the development set, 44 children in the testing set) | 26 parameters from clinical findings and US, including demographic data (age, body mass index), presentation (symptom duration, chief complaint), laboratory investigations, Imaging data from Doppler US, time and location of presentation | Orchiectomy or orchidopexy: AUC of 0.95, sensitivity of 0.92, specificity of 0.89  [Cross validation] | No available code, dataset, or usable application. | Moderate |
| Lin 2023 [55] | To differentiate normal from abnormal/scarred kidneys using SPECT in pediatric patients | CNN | Institutional series (301 patients) | 3D SPECT, 2D Maximum Intensity Projections, 2.5D Maximum Intensity Projections | Differentiation normal from abnormal kidneys:  AUC:  3D - 0.91; 2.5D - 0.94; 2D(coronal) - 0.92; 2D(sagittal) - 0.87; 2D(transverse) - 0.92  [Holdout validation] | No available code, dataset, or usable application. | Moderate |
| Santori 2007 [56] | To predict delayed decrease in serum creatinine in pediatric kidney recipients | ANN | Institutional series (148 patients) | 20 variables (incl: patient demographics, early serum creatinine, urine volume, pre-transplant characteristics) | Predicting delayed increase in creatinine: AUC of 0.89, accuracy of 0.87    [Holdout validation] | Code is described (Visual Basic , C++) and may be available upon contact or readily generated on Statistica, no dataset or predictive tool. | Moderate |
| Tokar 2021 [57] | To predict enuresis in children | Logistic regression; also used Trees, Bayes, SVM, deep learning | Administrative dataset (8071 children) | 14 variables (clinical factors, urinary habits, family history, lower urinary tract symptoms) | Enuresis: AUC of 0.81, accuracy of 0.81    [Holdout validation] | No available code or dataset, no predictive tool. | Low |
| Wang 2021b [58] | To predict the time pediatric urologists require to complete a clinic visit | RF | Institutional series (256 visits) | Demographics, visit-level covariates (including diagnosis) | In-room doctor visit time: accurate to 3.6 minutes for new patients, and within 5 minutes for returning patients    [Holdout validation] | No available code or dataset, no predictive tool. | Moderate |
| Zheng 2019 [59] | To classify kidneys of normal children and those with CAKUT | SVM and CNN with transfer learning | Institutional series (100 children) | Features from segmented kidneys by transfer learning and conventional imaging | CAKUT (bilateral, right, left), AUC between 0.92, accuracy between 0.81 and 0.87    [Cross validation] | Extensive model description, available dataset, no available code or predictive tool. | Moderate |

**Abbreviations**

ANN Artificial neural network

AUC Area under the receiver operator characteristic

CNN Convolutional neural network

EBC Estimated bladder capacity

LDA Linear Discriminant Analysis

MSE Mean squared error

NME Normalized mean error
RF Random forest

SVM Support vector machines

UPJO Ureteropelvic junction obstruction

US Ultrasound

UTI Urinary tract infection

VUDS Video urodynamics

**References**

[1] BAGLI DJ, AGARWAL SK, VENKATESWARAN S, SHUCKETT B, KHOURY AE, MERGUERIAN PA, et al. Artificial neural networks in pediatric urology: prediction of sonographic outcome following pyeloplasty. J Urol 1998;160:980–3.

[2] Blum ES, Porras AR, Biggs E, Tabrizi PR, Sussman RD, Sprague BM, et al. Early Detection of Ureteropelvic Junction Obstruction Using Signal Analysis and Machine Learning: A Dynamic Solution to a Dynamic Problem. J Urol 2018;199:847–52. https://doi.org/10.1016/j.juro.2017.09.147.

[3] Cerrolaza JJ, Peters CA, Martin AD, Myers E, Safdar N, Linguraru MG. Quantitative ultrasound for measuring obstructive severity in children with hydronephrosis. J Urol 2016;195:1093–9.

[4] Dhindsa K, Smail LC, McGrath M, Braga LH, Becker S, Sonnadara RR. Grading prenatal hydronephrosis from ultrasound imaging using deep convolutional neural networks. 2018 15th Conf. Comput. Robot Vis., IEEE; 2018, p. 80–7.

[5] Drysdale E, Khondker A, Kim JK, Kwong JCC, Erdman L, Chua M, et al. Personalized application of machine learning algorithms to identify pediatric patients at risk for recurrent ureteropelvic junction obstruction after dismembered pyeloplasty. World J Urol 2022;40:593–9.

[6] Erdman L, Skreta M, Rickard M, McLean C, Mezlini A, Keefe DT, et al. Predicting Obstructive Hydronephrosis Based on Ultrasound Alone. Lect. Notes Comput. Sci. (including Subser. Lect. Notes Artif. Intell. Lect. Notes Bioinformatics) - Med. Image Comput. Comput. Assist. Interv. – MICCAI 2020, vol. 12263 LNCS, Springer Science and Business Media Deutschland GmbH; 2020, p. 493–503. https://doi.org/10.1007/978-3-030-59716-0_47.

[7] Guan Y, Wen P, Li J, Ma Z. A DWT-Utilized Classifier for UPJO Diagnosis Using Ultrasound Images. 2022 IEEE Int. Conf. Networking, Sens. Control, IEEE; 2022, p. 1–6.

[8] Khondker A, Kwong JCC, Chancy M, D’Souza N, Kim K, Kim JK, et al. Obstruction risk from common ultrasound parameters in pediatric hydronephrosis with machine learning. BJU Int 2023.

[9] Lorenzo AJ, Rickard M, Braga LH, Guo Y, Oliveria JP. Predictive Analytics and Modeling Employing Machine Learning Technology: The Next Step in Data Sharing, Analysis, and Individualized Counseling Explored With a Large, Prospective Prenatal Hydronephrosis Database. Urology 2019;123:204–9. https://doi.org/10.1016/j.urology.2018.05.041.

[10] Ostrowski DA, Logan JR, Antony M, Broms R, Weiss DA, Van Batavia J, et al. Automated Society of Fetal Urology (SFU) grading of hydronephrosis on ultrasound imaging using a convolutional neural network. J Pediatr Urol 2023.

[11] Roshanitabrizi P, Zember J, Sprague BM, Hoefer S, Sanchez-Jacob R, Jago J, et al. Standardized analysis of kidney ultrasound images for the prediction of pediatric hydronephrosis severity. Mach. Learn. Med. Imaging 12th Int. Work. MLMI 2021, Held Conjunction with MICCAI 2021, Strasbourg, Fr. Sept. 27, 2021, Proc. 12, Springer; 2021, p. 366–75.

[12] Sloan M, Li H, Lescay HA, Judge C, Lan L, Hajiyev P, et al. Pilot study of machine learning in the task of distinguishing high and low-grade pediatric hydronephrosis on ultrasound. Investig Clin Urol 2023;64:588–96. https://doi.org/https://dx.doi.org/10.4111/icu.20230170.

[13] Seçkiner I, Seçkiner SU, Bayrak Ö, Erturhan S. Use of artificial neural networks in the management of antenatally diagnosed ureteropelvic junction obstruction. Can Urol Assoc J 2011;5:E152.

[14] Smail LC, Dhindsa K, Braga LH, Becker S, Sonnadara RR. Using Deep Learning Algorithms to Grade Hydronephrosis Severity: Toward a Clinical Adjunct. Front Pediatr 2020;8. https://doi.org/10.3389/fped.2020.00001.

[15] Tabrizi PR, Zember J, Sprague BM, Hoefer S, Sanchez-Jacob R, Jago J, et al. Pediatric hydronephrosis severity assessment using convolutional neural networks with standardized ultrasound images. 2021 IEEE 18th Int. Symp. Biomed. Imaging, IEEE; 2021, p. 1803–6.

[16] Tsai M-C, Lu HH-S, Chang Y-C, Huang Y-C, Fu L-S. Automatic screening of pediatric renal ultrasound abnormalities: deep learning and transfer learning approach. JMIR Med Informatics 2022;10:e40878.

[17] Weaver JK, Logan J, Broms R, Antony M, Rickard M, Erdman L, et al. Deep Learning of Renal Scans in Children with Antenatal Hydronephrosis. J Pediatr Urol 2023.

[18] Abbas TO, AbdelMoniem M, Chowdhury MEH. Automated quantification of penile curvature using artificial intelligence. Front Artif Intell 2022;5:954497.

[19] Abbas TO, AbdelMoniem M, Khalil IA, Hossain MSA, Chowdhury MEH. Deep learning based automated quantification of urethral plate characteristics using the plate objective scoring tool (POST). J Pediatr Urol 2023;19:373-e1.

[20] Baray SB, Abdelmoniem M, Mahmud S, Kabir S, Faisal MAA, Chowdhury MEH, et al. Automated measurement of penile curvature using deep learning-based novel quantification method. Front Pediatr 2023;11:1149318.

[21] Fernandez N, Lorenzo AJ, Rickard M, Chua M, Pippi-Salle JL, Perez J, et al. Digital pattern recognition for the identification and classification of hypospadias using artificial intelligence vs experienced pediatric urologist. Urology 2021;147:264–9.

[22] He Z, Yang B, Tang Y, Wang X. Development and verification of machine learning model based on anogenital distance, penoscrotal distance, and 2D: 4D finger ratio before puberty to predict hypospadias classification. Front Pediatr 2024;12:1297642.

[23] Li M, Wang Q, Lu P, Zhang D, Hua Y, Liu F, et al. Development of a machine learning-based prediction model for chemotherapy-induced myelosuppression in children with wilms’ tumor. Cancers (Basel) 2023;15:1078.

[24] Ma X-H, Shu L, Jia X, Zhou H-C, Liu T-T, Liang J-W, et al. Machine learning-based CT radiomics method for identifying the stage of Wilms tumor in children. Front Pediatr 2022;10:873035.

[25] Sharaby I, Alksas A, Nashat A, Balaha HM, Shehata M, Gayhart M, et al. Prediction of wilms’ tumor susceptibility to preoperative chemotherapy using a novel computer-aided prediction system. Diagnostics 2023;13:486.

[26] Song H, Wang X, Wang H, Guo F, Wu R, Liu W. The application of machine learning based on computed tomography images in the identification of renal tumors in children. Transl Pediatr 2024;13:417.

[27] Zhu Y, Li H, Huang Y, Fu W, Wang S, Sun N, et al. CT-based identification of pediatric non-Wilms tumors using convolutional neural networks at a single center. Pediatr Res 2023;94:1104–10. https://doi.org/https://dx.doi.org/10.1038/s41390-023-02553-x.

[28] Abdovic S, Cuk M, Cekada N, Milosevic M, Geljic A, Fusic S, et al. Predicting posterior urethral obstruction in boys with lower urinary tract symptoms using deep artificial neural network. World J Urol 2019;37:1973–9.

[29] Kwong JCC, Khondker A, Kim JK, Chua M, Keefe DT, Dos Santos J, et al. Posterior Urethral Valves Outcomes Prediction (PUVOP): a machine learning tool to predict clinically relevant outcomes in boys with posterior urethral valves. Pediatr Nephrol 2021:1–8.

[30] Yin S, Peng Q, Li H, Zhang Z, You X, Fischer K, et al. Multi-instance deep learning of ultrasound imaging data for pattern classification of congenital abnormalities of the kidney and urinary tract in children. Urology 2020;142:183–9.

[31] Arlen AM, Alexander SE, Wald M, Cooper CS. Computer model predicting breakthrough febrile urinary tract infection in children with primary vesicoureteral reflux. J Pediatr Urol 2016;12:288-e1.

[32] Bertsimas D, Li M, Estrada C, Nelson C, Scott Wang H-H. Selecting Children with Vesicoureteral Reflux Who are Most Likely to Benefit from Antibiotic Prophylaxis: Application of Machine Learning to RIVUR. J Urol 2021;205:1170–9.

[33] Dubrov VI, Sizonov V V, Kagantsov IM, Negmatova KN, Bondarenko SG. Predicting the outcomes of a single endoscopic correction of vesicoureteral reflux using a dextranomer/hyaluronic acid copolymer: selection of the optimal predictive model. Urol Her 2021;9:45–55.

[34] Eroglu Y, Yildirim K, Çinar A, Yildirim M. Diagnosis and grading of vesicoureteral reflux on voiding cystourethrography images in children using a deep hybrid model. Comput Methods Programs Biomed 2021;210:106369.

[35] Ganapathy S, KT H, Jindal B, Naik PS, Nair N S. Comparison of diagnostic accuracy of models combining the renal biomarkers in predicting renal scarring in pediatric population with vesicoureteral reflux (VUR). Irish J Med Sci 2023;192:2521–6.

[36] Kabir S, Salle JLP, Chowdhury MEH, Abbas TO. Quantification of vesicoureteral reflux using machine learning. J Pediatr Urol 2024;20:257–64.

[37] Keskinoglu A, Ozgur S. The use of artificial neural networks for differential diagnosis between vesicoureteral reflux and urinary tract infection in children 2020.

[38] Khondker A, Kwong JCC, Rickard M, Skreta M, Keefe DT, Lorenzo AJ, et al. A machine learning-based approach for quantitative grading of vesicoureteral reflux from voiding cystourethrograms: Methods and proof of concept. J Pediatr Urol 2021.

[39] Khondker A, Kwong JCC, Yadav P, Chan JYH, Singh A, Skreta M, et al. Multi-institutional validation of improved vesicoureteral reflux assessment with simple and machine learning approaches. J Urol 2022;208:1314–22.

[40] Knudson MJ, Austin JC, Wald M, Makhlouf AA, Niederberger CS, Cooper CS. Computational model for predicting the chance of early resolution in children with vesicoureteral reflux. J Urol 2007;178:1824–7.

[41] Li Z, Tan Z, Wang Z, Tang W, Ren X, Fu J, et al. Development and multi-institutional validation of a deep learning model for grading of vesicoureteral reflux on voiding cystourethrogram: a retrospective multicenter study. Eclinicalmedicine 2024;69.

[42] Logvinenko T, Chow JS, Nelson CP. Predictive value of specific ultrasound findings when used as a screening test for abnormalities on VCUG. J Pediatr Urol 2015;11:176-e1.

[43] Seckiner I, Seckiner SU, Erturhan S, Erbagci A, Solakhan M, Yagci F. The use of artificial neural networks in decision support in vesicoureteral reflux treatment. Urol Int 2008;80:283–6.

[44] Serrano‐Durbá A, Serrano AJ, Magdalena JR, Martín JD, Soria E, Domínguez C, et al. The use of neural networks for predicting the result of endoscopic treatment for vesico‐ureteric reflux. BJU Int 2004;94:120–2.

[45] Estrada CR, Nelson CP, Wang HH, Bertsimas D, Dunn J, Li M, et al. Targeted workup after initial febrile urinary tract infection: Using a novel machine learning model to identify children most likely to benefit from voiding cystourethrogram. J Urol 2019;202:144–52. https://doi.org/10.1097/JU.0000000000000186.

[46] Lee H, Yoo B, Baek M, Choi JY. Prediction of Recurrent Urinary Tract Infection in Paediatric Patients by Deep Learning Analysis of 99mTc-DMSA Renal Scan. Diagnostics 2022;12:424.

[47] Wang H-HS, Li M, Cahill D, Panagides J, Logvinenko T, Chow J, et al. A machine learning algorithm predicting risk of dilating VUR among infants with hydronephrosis using UTD classification. J Pediatr Urol 2024;20:271–8.

[48] Ge Z, Tang L, Peng Y, Zhang M, Tang J, Yang X, et al. Design of a rapid diagnostic model for bladder compliance based on real-time intravesical pressure monitoring system. Comput Biol Med 2022;141:105173.

[49] Hobbs KT, Choe N, Aksenov LI, Reyes L, Aquino W, Routh JC, et al. Machine Learning for Urodynamic Detection of Detrusor Overactivity. Urology 2022;159:247–54.

[50] Yuan G, Ge Z, Zheng J, Yan X, Fu M, Li M, et al. CNN-based diagnosis model of children’s bladder compliance using a single intravesical pressure signal. Comput Methods Biomech Biomed Engin 2024:1–12.

[51] Wang HS, Cahill D, Panagides J, Nelson CP, Wu H, Estrada C. Pattern recognition algorithm to identify detrusor overactivity on urodynamics. Neurourol Urodyn 2021;40:428–34.

[52] Weaver JK, Martin-Olenski M, Logan J, Broms R, Antony M, Van Batavia J, et al. Deep Learning of Videourodynamics to Classify Bladder Dysfunction Severity in Patients With Spina Bifida. J Urol 2023;209:994–1003. https://doi.org/https://dx.doi.org/10.1097/JU.0000000000003267.

[53] Çelik Ö, Aslan AF, Osmanoğlu UÖ, Cetın N, Tokar B. Estimation of renal scarring in children with lower urinary tract dysfunction by utilizing resampling technique and machine learning algorithms. J Surg Med 2020;4:573–7.

[54] Ekşi M, Yavuzsan AH, Evren İ, Ayten A, Fakir AE, Akkaş F, et al. Testicular salvage: using machine learning algorithm to develop a predictive model in testicular torsion. Pediatr Surg Int 2022;38:1481–6.

[55] Lin C, Chang Y-C, Chiu H-Y, Cheng C-H, Huang H-M. Differentiation between normal and abnormal kidneys using 99mTc-DMSA SPECT with deep learning in paediatric patients. Clin Radiol 2023;78:584–9.

[56] Santori G, Fontana I, Valente U. Application of an artificial neural network model to predict delayed decrease of serum creatinine in pediatric patients after kidney transplantation. Transplant. Proc., vol. 39, Elsevier; 2007, p. 1813–9.

[57] Tokar B, Baskaya M, Celik O, Cemrek F, Acikgoz A. Application of Machine Learning Techniques for Enuresis Prediction in Children. Eur J Pediatr Surg 2021;31:414–9. https://doi.org/10.1055/s-0040-1715655.

[58] Wang H-HS, Cahill D, Panagides J, Yang T-YT, Finkelstein J, Campbell J, et al. A Machine Learning Model to Maximize Efficiency and Face Time in Ambulatory Clinics. Urol Pract 2021;8:176–82.

[59] Zheng Q, Furth SL, Tasian GE, Fan Y. Computer-aided diagnosis of congenital abnormalities of the kidney and urinary tract in children based on ultrasound imaging data by integrating texture image features and deep transfer learning image features. J Pediatr Urol 2019;15:75-e1.
