# Supplementary material for: A living scoping review and online repository of artificial intelligence models in pediatric urology: Results from the AI-PEDURO collaborative
Source: J Pediatr Urol. Author manuscript; Available in PMC 2026 Feb 16. (PMC12908176; doi:10.1016/j.jpurol.2025.01.035)
Supplement: Supplemental mmc1 [file NIHMS2144933-supplement-Supplemental_mmc1.docx]

**Supplementary Figure 1:** Concept of AI-PEDURO, where (left) an annual living scoping review is conducted to input into the repository, and (right) user-submitted models reviewed monthly are assessed for inclusion into the repository.

**Supplementary Figure 2**: Mean APPRAISE-AI item scores for all studies using AI in pediatric urology. Each field is presented as a percentage of the maximum possible score for that field (i.e., mean score/maximum possible score x 100%) to compare scores between fields, irrespective of the assigned weighting. Items are coloured based on percentage of their corresponding maximum possible score: red for less than 40%, blue for between 40 and 60%, and green for greater than or equal to 60%.


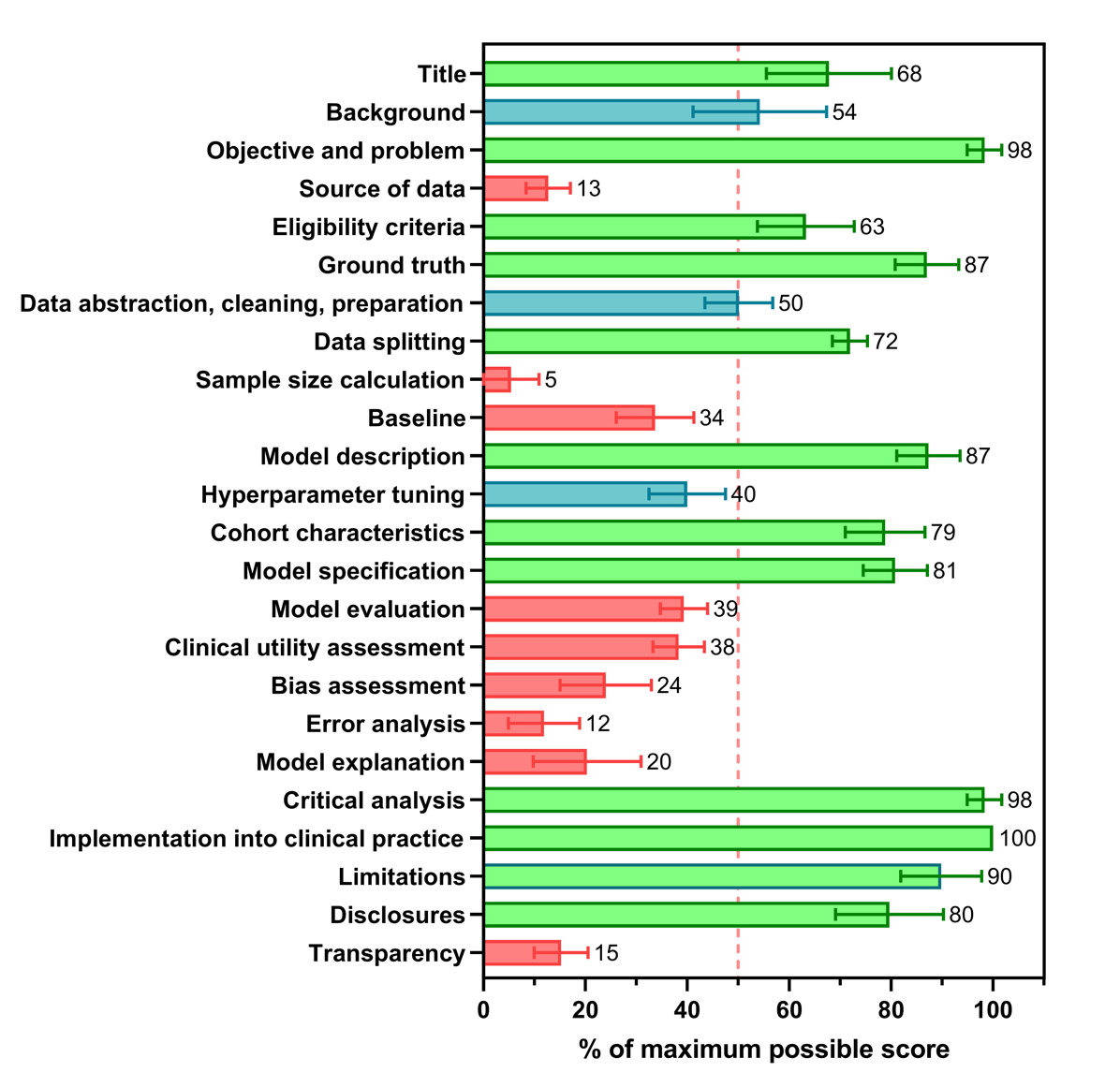


**Supplementary Table 1:** Search Strategy from Embase Classic+Embase 1947 to 2024 June 21.

| **#** | **Searches** | **Results** |
| --- | --- | --- |
| 1 | exp child/ or exp pediatrics/ or child*.ti,ab. or infan*.ti,ab. or (baby or babies).ti,ab. or exp adolescent/ or adolescen*.ti,ab. or (pediatric*1 or paediatric*1).ti,ab. or (neonat* or newborn*).ti,ab. | 5607953 |
| 2 | exp urology/ or exp child urology/ | 53835 |
| 3 | exp hydronephrosis/ or exp vesicoureteral reflux/ | 47658 |
| 4 | exp urodynamics/ or exp obstructive uropathy/ | 72909 |
| 5 | exp spinal dysraphism/ | 16851 |
| 6 | exp urinary tract infection/ | 158349 |
| 7 | exp pyeloplasty/ | 5215 |
| 8 | exp nephroblastoma/ | 21337 |
| 9 | exp hypospadias/ | 13272 |
| 10 | exp urolithiasis/ or exp nephrolithiasis/ | 85029 |
| 11 | exp artificial intelligence/ or exp machine learning/ | 527258 |
| 12 | (neural network or support vector machine or multilayer perceptron or neural network or random forest or lasso or ridge or kernel or bayesian network or classification tree or regression tree or vector machine or nearest neighbor or probability estimation tree or elastic net or ensemble or penalized or regularized or bagging or boosting or fuzzy or bayes or deep learning).ti,ab. | 358863 |
| 13 | 1 and (2 or 3 or 4 or 5 or 6 or 7 or 8 or 9 or 10) and (11 or 12) | 473 |

**Supplementary Table 2:** Example model card for Khondker et al. (2023) [5]; yellow rows are provided for all models, and green rows are optionally provided.

| **Article DOI** | https://doi.org/10.1111/bju.16159 |
| --- | --- |
| **Objective** | To predict the risk of renal obstruction on diuretic renography using routine reported ultrasonography findings |
| **AI Approach** | Random Forest Model (multi-class classification) |
| **Data Source(s)** | *Internal*: 304 patients (The Hospital for Sick Children)  *Validation*: 64 patients (Children’s Hospital of Philadelphia) |
| **Model Input** | Age, Sex, Kidney laterality, Kidney length, Anteroposterior diameter, SFU grade |
| **Model Outcome** | T1/2 time on diuretic renogram (<20 min, 20-60 min, >60 min) |
| **Model Metrics** | Multi-class AUROC = 0.75  Binary AUROC (>20 min) = 0.84 |
| **Model Usability** | https://sickkidsurology.shinyapps.io/AERO/ |
| **Model Quality** | Very High Quality |
| **Lay Summary** | This model predicts the likelihood of blockage in children with swollen kidneys on a nuclear medicine scan, which may influence whether a nuclear medicine scan is needed |
